# Dynamic optimization and conformity in health behavior and life enjoyment over the life cycle

**DOI:** 10.3389/fnbeh.2015.00137

**Published:** 2015-06-16

**Authors:** Hernán D. Bejarano, Hillard Kaplan, Stephen Rassenti

**Affiliations:** ^1^Economic Science Institute, Chapman UniversityOrange, CA, USA; ^2^CIDE, Center for Economics Reserach and TeachingAguascalientes, Mexico; ^3^Anthropology, University of New MexicoAlbuquerque, NM, USA

**Keywords:** behavioral economics, health economics, experimental economics, health knowledge, attitudes, practice, dynamics programming

## Abstract

This article examines individual and social influences on investments in health and enjoyment from immediate consumption. Our lab experiment mimics the problem of health investment over a lifetime (Grossman, 1972a,b). Incentives to find the appropriate expenditures on life enjoyment and health are given by making in each period come period a function of previous health investments. In order to model social effects in the experiment, we randomly assigned individuals to chat/observation groups. Groups were permitted to freely chat between repeated lifetimes. Two treatments were employed: In the Independent-rewards treatment, an individual's rewards from investments in life enjoyment depend only on his choice and in the Interdependent-rewards treatment; rewards not only depend on an individual's choices but also on their similarity to the choices of the others in their group, generating a premium on conformity. *The principal hypothesis is that gains from conformity increase variance in health behavior among groups and can lead to suboptimal performance*. We tested three predictions and each was supported by the data: the Interdependent-rewards treatment (1) decreased within-group variance, (2) increased between-group variance, and (3) increased the likelihood of behavior far from the optimum with respect to the dynamic problem. We also test and find support for a series of subsidiary hypotheses. We found: (4) Subjects engaged in helpful chat in both treatments; (5) there was significant heterogeneity among both subjects and groups in chat frequencies; and (6) chat was most common early in the experiment, and (7) the interdependent rewards treatment increased strategic chat frequency. Incentives for conformity appear to promote prosocial behavior, but also increase variance among groups, leading to convergence on suboptimal strategies for some groups. We discuss these results in light of the growing literature focusing on social networks and health outcomes.

## Introduction

Inequalities in health, across and within populations, are a major public concern, but the causal processes underlying social effects on health remain incompletely understood and subject to much debate (see, for example, Weinstein and Lane, 2014 for a recent collection of papers commissioned by the National Academy Sciences to address those processes). There are large ethnic differences in both life expectancy within the U.S. For example, in the 1990s the life expectancy of Native American males was 56 years in some counties, while that of Asian American women was 95 years in other counties (Murray et al., 1999). While several critical variables such as income and education help explain these differences, significant variance remains unexplained (Cutler and Lleras-Muney, 2010). There are significant national differences in health-related behavior, such as obesity (Finucane et al., 2011) and those differences vary by gender (Kanter and Caballero, 2012). There are also regional differences in the U.S. with obesity being greater in the central states compared to the east and west coasts (Kelley et al., 2015). There are also ethnic differences that interact with socio-economic status and sex (Jackson et al., 2013; Hernandez and Pressler, 2014). In fact, health-related behaviors such as exercise, dietary habits, and smoking and alcohol consumption, have been calculated to explain about 40–50% percent of premature mortality, as well as substantial morbidity and disability, in the United States (McGinnis et al., 2002).

Empirical evidence shows that social groups influence health behavior in complex ways. Peer pressure can help individuals control health habits (Umberson et al., 2010). For example, spouses or religious communities may monitor, inhibit, regulate, or facilitate the health behavior of their partners or members of their community (Waite, 1995; Ellison and Levin, 1998). Group effects alternatively might lead individuals to engage in risk-taking and increased alcohol consumption. In addition, there seem to be matching effects; for example, having an obese spouse or friend can increase an individual's likelihood of being obese (Crosnoe et al., 2004; Christakis and Fowler, 2007). There is a growing body of literature concerned with the way in which social networks influence health-related behavior (Crosnoe et al., 2004; Christakis and Fowler, 2007; Hutchinson and Rapee, 2007; Ajilore et al., 2014; Girard et al., 2014; Shimizu et al., 2014; Tucker et al., 2014; Nam et al., 2015). Yet, despite the extensive evidence of group influence in health behavior, little is known of the precise mechanisms by which groups influence individual choices (Cruwys et al., 2015). Identifying the social causes of behavioral change from naturally occurring data is difficult due to selection biases and unobserved heterogeneity associated with group formation (Fowler et al., 2011). In addition, interactions between individuals and groups that affect health behavior are usually unobserved.

The principal hypothesis motivating the experiments this paper reports is that *social influences increase variance in health behavior among groups, resulting in these “cultural” differences in health*. We propose that social effects derive from two principle routes. First, people utilize observation of behavior and engage in direct communication about practices and strategies in order to be better able to achieve their goals. Providing advice and educating others is an intrinsically human and pro-social activity. Humans have been providing advice regarding health behavior for millennia (Kleinman, 1980), and now they can even provide advice to strangers on the Internet (Constant et al., 1996; Swan, 2012).

A second route for social effects derives from the increased utility people gain by the extent to which their choices conform to those of others, with whom they interact and identify. We propose that this route is a major driver producing variance among groups. Many of the activities that can compromise health, such as smoking and consuming large amounts of food or alcohol, are often done in group contexts (Pliner and Mann, 2004; Blake et al., 2011). This is why weight gain is particularly concentrated during the end-of-year holiday season (Yanovski et al., 2000). We propose that individuals, who refrain from such group activities for health reasons, when they are common in their social network, suffer social costs, both because they are rejecting the social norm and because they are forgoing opportunities to invest in affiliative social bonds. For example, a large meta-analysis of 69 eligible experiments showed strong evidence that eating behavior is modeled in social groups, and that modeling is enhanced when individual desire to affiliate with the model. Alcohol consumption in groups has been shown to increase social bonding (Sayette et al., 2012), forays to local bars for communal drinking are common in many cultural and occupational groups (e.g., Duke et al., 2013; Pillai et al., 2013). Our hypothesis is that socio-cultural variation in health outcomes is partially driven by conformity biases is these kinds of health-related behaviors, and that those biases will amplify variation among groups.

To test this hypothesis, this article examines individual and social influences on investments in health and enjoyment from immediate consumption in two group contexts. We do this in a specially framed lab experiment that mimics the problem of health investment over a lifetime, building on Grossman's (1972a,b) theoretical framework to study health investment choices. Choosing optimal health investments over the life course is a complex task. Individuals might estimate well the current costs and benefits of their actions but be less certain of their long-run effects. In essence, to determine how much time, income and effort to invest in healthy behavior, individuals have to solve a dynamic programing problem addressing uncertainty concerning future income and progressive health degeneration.

In the lab, our subjects experienced an experimental environment that mimics the previously described health-investment problem. Each subject lives a nine-period life, during each period of which he earns income and then invests some proportion of that income in health and some in life enjoyment. Subjects earn money in the experiment in proportion to the sum of the life enjoyment they have consumed. However, income in each period of life is a function of previous health investments, so there is a dynamic optimum achievable that maximizes earnings (aggregate life enjoyment) through the appropriate investments in life enjoyment and health in each period. Subjects live eight separate lives during the experiment with identical parameter values in each life, so they can learn from experience.

In order to model social effects in the experiment, we randomly assigned individuals to chat/observation groups, composed of four subjects each. Between lives, subjects were allowed to chat with and observe the choices of others in their chat group. We employed the chat room discussions during the experiments to study how advice and queries about the appropriate investment strategies affected behavior. Subjects in our experiment, just as individuals in real life, are never isolated in their ability to compare and assess the quality of their individual decisions. Our experimental approach in which chat/observation groups are formed randomly and in which interactions between the individuals of the groups are recorded, allows us to analyze whether a mechanism exists that links health behavior and group communication.

Our experimental design presents two treatments to investigate social impacts on health. In both treatments, individual subjects are embedded in social groups of four individuals that do not change composition throughout the entire experiment (8 lives). Subjects in both treatments can observe the choices made by others in their social group and are permitted to chat with fellow group members. The difference between the two treatments derives from the relationship between investments in life enjoyment and experimental earnings. In our baseline *Independent-Rewards* treatment, an individual's rewards from investments in life enjoyment depend only on his choices. When rewards are independent of others' choices, individuals do not have monetary incentives to provide any advice. However, individuals still have an incentive to search for advice, and particularly, might be willing to post queries about strategies, hoping that those who perform better will voluntarily provide some guidance. Therefore, in the independent-rewards treatment, an individual's willingness to provide advice is mostly generated by their intrinsic motivation to help others. This mimics the first “purely informational” cultural route.

In the second *Interdependent-Rewards* treatment, intended to mimic environments where strong social norms are impinging on decision-making, rewards not only depend on an individual's choices but also on their similarity to the choices of the others in their group. Individuals have a payoff function that provides them incentive to make behavioral choices similar to the other members of their group (a conformity coefficient). Therefore, in the interdependent treatment, individuals have a monetary extrinsic motivation to discuss, agree, and coordinate on life enjoyment investment, and its correlated investment in health. The form of this monetary extrinsic motivation is perfectly known to each subject: a graph shows her precisely how much her life enjoyment will decrease as the difference between what she and others in her group invest increases. It is important to note that the parameters of the experiment were designed such that the theoretically optimal sequence of decisions was identical in both treatments, suggesting that any systematic differences in outcomes observed are attributable to the need for social coordination in the interdependent treatment and the imperative nature of the extrinsic motivation to advice. It is possible to imagine that strong social norms could at times overwhelm the general health initiative and lead a group to a conforming but sub-optimal outcome.

This second route may reinforce the optimizing effects of the first route, but may also lead to multiple equilibria. In other words, communication, queries and advice regarding health behavior, can improve health investment and life-enjoyment choices, but also can lead to suboptimal equilibrium habits.

The central predictions we test in this paper are:

1. The conformity payoff in the interdependent-rewards treatment will *decrease* within-group variance in behavior.
2. Due to the possibility of multiple equilibria, the interdependent treatment will *increase* among-group variance in behavior.
3. The likelihood of poor performance, in terms of optimizing investments per period over the life course, will be greater in the interdependent-rewards treatment.
  We also test the following subsidiary predictions derived from the first purely informational route and the added incentives to communicate when investment rewards are interdependent in the conformity treatment.
4. In both treatments, subjects will make queries and provide strategic advice during chat.
5. Significant chat heterogeneity will exist between groups, above the individual heterogeneity of its members, through processes of observation and information exchange.
6. Advice and queries will be most common during the first few lives of the experiment while individuals are most focused on learning.
7. Due to incentives, chat about investment behavior will be more frequent in the interdependent treatment than in the independent treatment.

The paper is organized as follows. In Section The Health Investment Problem of this paper we describe the health investment problem precisely as it is presented to the individuals in our experimental environment. In Subsection Experimental Environment and Corresponding Theory we illustrate the decision making task faced by the subjects and outline the dynamic optimization problem the individual must solve in order to maximize his aggregate life enjoyment which is linearly proportional to what he gets paid to participate. In Subsection Multiple Lives and Chat Groups we discuss the group structure imposed on subjects and the treatment variable distinguishing group social conditions. In Section Statistical Approach we briefly lay out the data structure used to support our statistical analyses. Section Descriptive Statistics provides descriptive statistics of the data collected while Section Predictors of Enjoyment Earnings and Assessment of Social Effects analyzes predictors of enjoyment earnings and assesses social effects. Section Discussion and Conclusions discusses the results found in light of the introduction and intent of this study.

## The health investment problem

### Experimental environment and corresponding theory

In the experiments each individual participant worked at a real-effort harvesting task to earn income and made a sequence of health and life enjoyment investment decisions in a series of unrelated lifetimes. Each lifetime was comprised of a sequence of 9 periods (*t* = 1, 2, … 9) of real-effort harvesting activity followed by investment decision- making. Every lifetime ended after nine periods, unless the participant's “health” had degenerated to the point of death before then, and the subject's earnings for that particular lifetime were computed and added to their cumulative experimental earnings. After each lifetime ended, every participant began a new life, until she along with the other members of group had completed a sequence of 8 lives.

**Figure 1:**
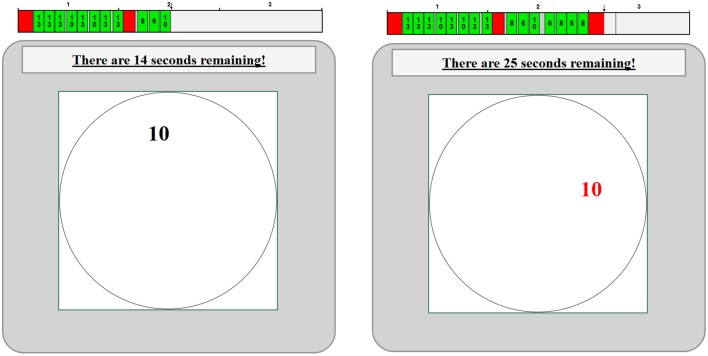
**Harvesting environment**.

The real effort harvesting task consisted of observing a circular harvesting field in which a new target would materialize at the center every second, and capturing the target presented using a mouse click before it skirted across the boundary. The sequence of targets varied randomly in value, and once a target was captured it would take 2 s to process it during which time the next target that came by was not available for harvesting. At the beginning of each period subjects also forfeited harvesting time in proportion to 1 minus their current state of health. The two similar figures displayed in Figure 1 represents what subjects might have seen during periods 2 and 3 at times when they could and could not harvest the current target worth 10 that was skirting across the harvest field.

Notice the bar charts above the two sample harvest fields. Each green element indicates a target captured and its value. Red spaces indicate times when the subject was unable to harvest, and gray indicates inactive or future time.

**Figure 2:**
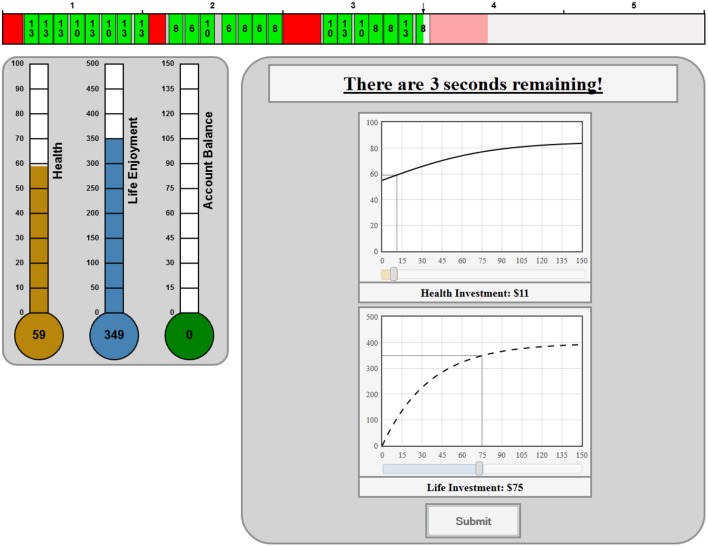
**Investment stage environment**.

Once the participant finished the real effort harvesting task in period t, from which effort she had secured harvest revenue^1^, R_t_, proportional to current health, she was required to make investment decisions: how much to invest, I_t_, in preserving health for future harvesting, how much to invest in life enjoyment, L_t_, in order to be paid for her efforts, and how much (if any) to leave uninvested in a bank account, B_t_, that would become available for future investments in life enjoyment or health. Figure 2 shows the screen that the subject might have seen after harvesting when it was time to make her individual investment decisions:

The total amount of revenue that the subject had to invest ($86) was comprised of her harvesting revenue ($70) from the most recent period in addition to anything remain in her bank account ($16) from previous periods. There were two graphs depicting the rates of return on Health and Life investments and complementary scroll bars beneath each graph that never let the subject spend more than was available. As she moved the scroll bars the thermometers to the left of the graphs would indicate the state of Health and Life Enjoyment that would result from the given investments. For example, if this subject invested $11 in Health and $75 in Life Enjoyment her Health would increase to 59 (/100) and she would derive 349 units of Life Enjoyment with nothing left in her Bank account.

All participants were endowed with a beginning bank balance, B_0_, of 0, and should end with a final bank balance, B_9_, of 0, if they maximize their total gains from life enjoyment. The budget constraints governing investment in each period were given by:

$$I_{t}+L_{t}+B_{t}=B_{t-1}+R_{t}\;\forall t=1,2,\ldots 9$$

The precise non-linear return functions for investments in health and life enjoyment are given below. They were designed to have diminishing returns to scale, so that the optimal investment pattern across time would display properties similar to a Grossman model. The transition equation in our experimental system relating final health in period t (H_t_) to final health in the previous period (H_t − 1_), given an investment (I_t_) in preserving health, and a natural degeneration (d_t_) of health that occurred during period t, was given by:

$$H_{t}=Min\;\left[100,\;H_{t-1}-{\text{d}}_{t}+30\frac{1-e^{-0.025{\text{I}}_{\text{t}}}}{1+e^{-0.025{\text{I}}_{\text{t}}}}\right]$$

A participant could theoretically regenerate health by up to 30 points in any given period if she had accumulated an “infinite” amount of harvest revenue to invest, but an upper bound was imposed that prevented the next state of health from ever exceeding 100. Furthermore, the parameters in the experimental environment were chosen such that the boundary condition, H_t +1_ = 100, was never approached under optimal or “reasonable” decision making. Given the interior solution was always active, the marginal rate of return on health investment each period was given by:

$$\frac{dH_{t}}{dI_{t}}=\frac{1.5e^{-0.025{\text{I}}_{\text{t}}}}{1+2e^{-0.025{\text{I}}_{\text{t}}}+e^{-0.05{\text{I}}_{\text{t}}}}$$

Note that at I_t_ = 0, dH_t_/dI_t_ = 3/8 and the rate of return on each subsequent revenue unit invested in health is independent of initial state of health (H_t_) until health reaches 100. Over many periods and lifetimes, participants could become very familiar with the fixed function governing diminishing returns on health investment.

The earnings equation relating investment in life enjoyment (L_t_) to cash earned (E_t_) in period t, by a socially independent participant was given by:

$$\begin{array}{l} \text{Socially Independent Earnings}:\\ \;E_{t}=250\text{​​​ ​​}\left(1+H_{t}/100\right)\;\left(1-e^{-0.028L_{t}}\right) \end{array}$$

By convention, in any given period t, degradation of health occurred after harvesting. Then health investment, H_t_, selected was implemented prior to the life enjoyment investment, L_t_, so that the upgraded state of health would be incorporated into the life enjoyment computation. The participants were given graphical representations of the health and life enjoyment investments that made it very clear that both had diminishing returns^2^. The participant's job was to correctly balance investment of harvesting revenue between health and life enjoyment, each period of her lifetime. To maximize her earnings across her entire life (periods 1–9) the participant had to solve the following non-linear program:

$$\begin{array}{l} \text{ Maximize}:\sum_{t=1,9}E_{t}=\sum_{t=1,9}250(1+H_{t}/100)(1-{\text{e}}^{-0.028L_{t}})\\ \text{Subject to}:\;B_{t-1}+R_{t}=I_{t}+L_{t}\;\forall t=1,\ldots 9\\ \;H_{t}=H_{t-1}-{\text{d}}_{t}+30\frac{1-e^{-0.025{\text{I}}_{\text{t}}}}{1+e^{-0.025}\text{t}}\; \end{array}$$

                                                                        ∀ *t* = 1, … 9^3^

*R_t_* = *rev*(H_t_/100) during any active harvest period^4^.

**Figure 3:**
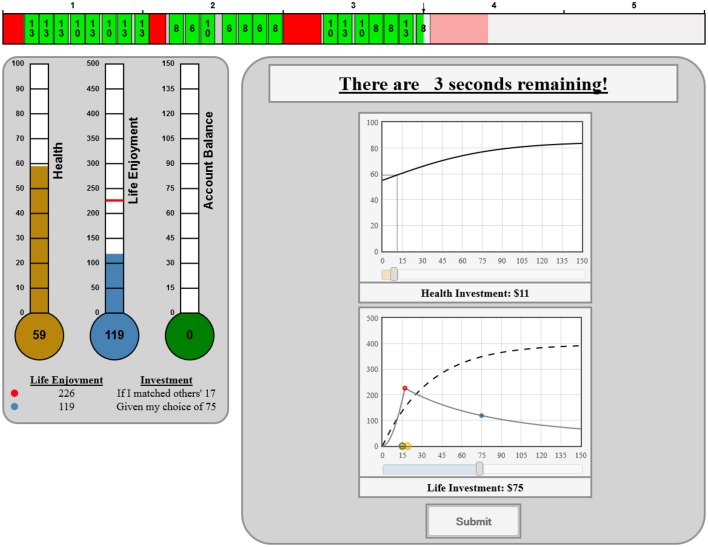
**Feedback stage interdependent treatment**.

The main treatment variable in the experiments reported determined whether each subject's earnings from investing in life enjoyment were interdependent or independent of the decisions made by other subjects in his social group. Figure 3 shows the feedback that a subject might see after the investment stage if she is part of a group whose life enjoyment is socially interdependent:

Notice the subject sees two lines on the Life Investment graph: the dotted line represents her return if she were socially independent and the solid line represents her return as a function of how close her life investment matches/matched the mean of the other three subjects in her group when she is socially interdependent. In experiments where subjects are interdependent subjects can adjust the location of the apex of the solid line according to their premonition concerning their group's mean investment. After investing they see the actual choices of others in their group and the resulting payment function. The legend under the thermometer gives subjects a verbal explanation of the outcome they observe.

The earnings equation for socially interdependent participants, relating investment in life enjoyment (L_t_) to cash earned (E_t_) in period t given the mean investment, O_t_, made by all other subjects in the subject's social group, is given by:

$$\begin{array}{l} \text{Socially Interdependent Earnings}:\\ E_{t}=1.5*\frac{Min\left(L_{t},O_{t}\right)}{Max\left(L_{t},O_{t}\right)}*\;250(1+H_{t}/100))(1-e^{-0.028L_{t}}) \end{array}$$

Note, these earnings were simply computed as the Socially Independent Earnings multiplied by a ‘conformity multiplier, $1.5*\frac{Min\left(L_{t},O_{t}\right)}{Max\left(L_{t},O_{t}\right)}$. The ratio $\frac{Min\left(L_{t},O_{t}\right)}{Max\left(L_{t},O_{t}\right)}$ measures the proportion by which the subject's life enjoyment investment, L_t_, matches the mean life enjoyment investment, O_t_, of other members of her social group. Under interdependence, a subject who conforms to the group mean in making her life investment choices could earn a premium of up to 50%, while one that strayed from her group's mean (more than 33% below or above) would find herself earning less than she would if she were not socially bound.

**Figure 4:**
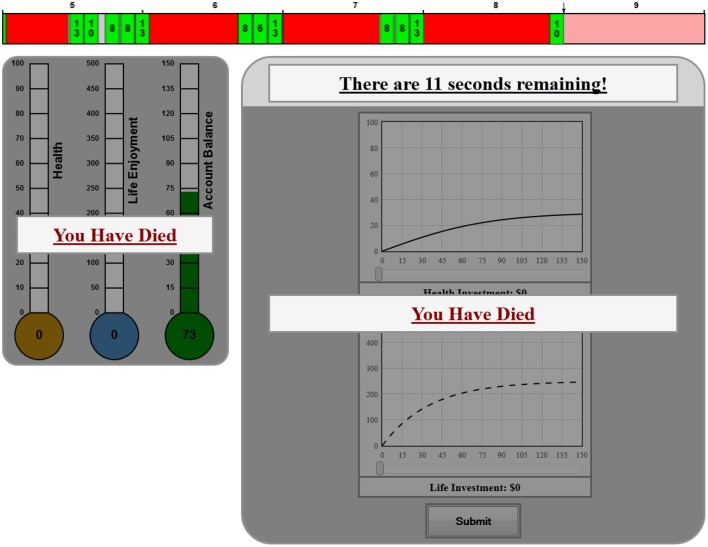
**Dead feedback screen**.

In our environments, the shape of the Health investment graph would never alter: only the starting point on the Y-axis, current health, would adjust from period to period. However, the shape of the Life Enjoyment Investment graph would get steeper if health deteriorated or flatten if health improved. If a subject made a poor sequence of investments in which she neglected maintaining her health, she could die prematurely before completing harvesting and investing through all nine periods of life (Figure 4). In such cases before the next harvesting period began the subject was informed of her inability to continue.

In the constrained dynamic maximization problem the subject must solve, H_t_ can be rewritten as a function of her initial health, H_0_, and investments, I_t_, in health.

$H_{t}=\;H_{0}+\sum_{k=1,t}-d_{k}+30\frac{1-e^{-0.025{\text{I}}_{\text{k}}}}{1+e^{-0.025{\text{I}}_{\text{k}}}}$ So solving the participant's constrained life enjoyment optimization can be rewritten as an unconstrained optimization that is a function of the sequence of health investments I_i_ and bank deposits B_t_^5^:

$$\begin{array}{l} \sum_{t=1,9}E_{t}=\text{​​}\sum_{t=1,9}250(1+\left[H_{0}+\sum_{k=1,t}-d_{k}+30\frac{1-e^{-0.025{\text{I}}_{\text{k}}}}{1+e^{-0.025{\text{I}}_{\text{k}}}}\right]\\ \;/100\left(1-e^{-0.028(B_{t-1}+R_{t}-I_{t})}\right) \end{array}$$

This problem is easy to solve numerically for any given period t when H_t − 1_, B_t − 1_, R_t_ and B_t_ are known^6^. The initial conditions for health and bank balance were given by H_0_ = 85 and B_0_ = 0, the final bank balance B_9_ must be zero, and R_t_ is always a linear function, rev(H_t − 1_/100), of previous period's health. We can either apply non-linear optimization or dynamic programming techniques to find the optimal sequence of health investments, I_t_, and the corresponding maximal aggregate life enjoyment ∑_*i* = 1,9_*E_t_*.

It is important to note that under Interdependence, even with its premium for investment conformity and penalty for non-conformity, the optimal investment pattern for like-skilled harvesters is exactly the same as it is under Independence Table 1 present the optimal investment in Health by period, these amounts are the same for all treatments. The best that any group can do is for all individuals to conform to what would otherwise be the optimal investment pattern for each under Independence: resulting earnings would simply be multiplied by 1.5.

**Table 1:** **Optimal Health (H_t_) by period**.

**1**	**2**	**3**	**4**	**5**	**6**	**7**	**8**	**9**
89	91	92	90	86	78	65	42	18

Using R_t_ = 87(H_t − 1_/100) (we found that 87 was the low variance, mean skill parameter of all participants), the period by period optimal Health (H_t_) profile that participants should maintain in order to make health investments (I_t_) that maximize total life enjoyment (∑E_t_) is given in the following table:

Table 2 table captures a quantitative representation of what is necessary to maintain this optimal health vector, and hints at some behavioral difficulties participants might encounter if their perception of optimal strategy requirements is less than perfect:

**Table 2:** **Optimal marginal rate of return, % of income invested in health, by period**.

**1**	**2**	**3**	**4**	**5**	**6**	**7**	**8**	**9**
10.0, 86	8.6, 80	7.4, 73	6.3, 67	5.3, 59	4.3, 50	3.3, 35	2.5, 0	2.5, 0

It shows the marginal rates of return for optimal investments in life enjoyment in each period of life, and implicitly the rate of return on investment in health and banking for current and future enjoyment maximization^7^. It also shows the percentage of income earned (plus banked^8^) that must be devoted to optimal health maintenance in each period of life.

Savvy participants must recognize that 86% of earned revenue from harvesting must be spent on health in period 1, and 80% in period 2, while the marginal rates of return on investments are 4 times larger than later in life: that skewed optimal investment strategy is a requirement to be reckoned with in splitting earned harvest revenues between health and life enjoyment. Late in life (periods 8 and 9), participants must let go of their health and spend entirely on life enjoyment. The complete solution for all decision and state variables are provided in Supplementary Material.

### Multiple lives and chat groups

This nine period dynamic optimization problem faced by subjects is difficult to solve, due to the non-linearities and interactions in the health and life enjoyment functions. In order to allow subjects to learn about the environment and to adjust their strategies accordingly, subjects lived eight nine-period lives under identical conditions. This “reincarnation” can be thought of as a way of modeling cultural traditions in which individuals learn from previous generations how to best perform in the environment. In addition, we proposed that in response to difficult dynamic problems, people would use observational learning and information exchange to help solve those problems. To model that process, subjects were divided in four-person chat-observation groups. Subjects could observe the behavior of three other subjects (the same three people in each life) and could chat with them, using text messages, between lives.

**Figure 5:**
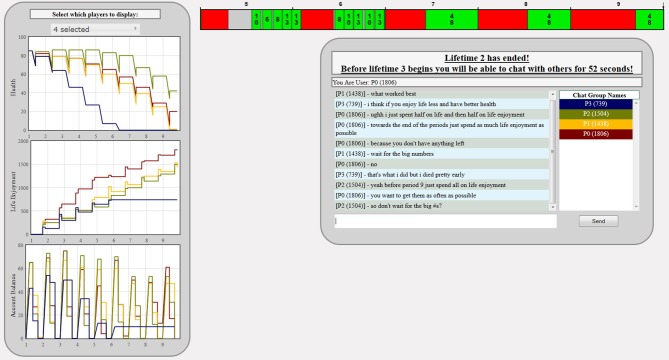
**Chat environment**.

Figure 5 shows the chat environment that subjects would see between lives:

Notice that the subjects could not only communicate with each other but could also select to see the entire set of actions (harvesting income, health and life investments period by period) for all or subjects in their group.

Under Social Independence the chat group provided nothing more than a venue to exchange information concerning individual strategy, but under Social Interdependence, the mean investment in Life Enjoyment by other members of a chat group became the norm by which investment of each group member was evaluated and translated into earnings. Interdependence allowed conformity in investment strategies to enhance earnings and non-conformity to penalize them. Under Interdependence, chat provided a venue for both optimizing and conforming strategies to evolve.

A total of 156 subjects were randomly allocated to 39 chat/observation groups: 68 subjects (17 chat groups) in the Independent treatment, 88 subjects (22 chat groups) in the Interdependent treatment. Members of each chat-group were free to observe and discuss (or not) each other's performances for 90 s at the end of each lifetime.

Subjects' conversations were captured by the messages written in chat window. Chat lines were classified independently by two independent research assistants that acted as coders^9^. Coders were trained to apply a classification criterion that captures the presence of strategic advice and queries.

To achieve this goal, coders classified lines into one of four thematic categories and into one of two linguistic categories. The thematic categories captured message's meaning, while the linguistic category captured the message's direction and intention. The four thematic categories were: Income Generation, Income Allocation, Other Experimental Issues, Non-Experimental chat. In this article we focus on the second category: this category includes all those messages in which subjects expressed ideas or concerns regarding the allocation of their income to health and life enjoyment. The two linguistic categories were: Statements or Queries. Chat lines were assigned to particular class only if both coders agreed on their classification.

A real example of chat observed over the experiment is displayed in Figure 5, in this example of dialogs between two subject P0 (810) requested advice, “*How much did you spend in health?,”* this chat line was codified by all of our coders as one belonging to the categories of, Income Allocation and Queries. Similarly, P2 (992) responded with two chat lines “*I just made sure my health level was around the same as it was in the beginning,” “and then I used all the rest on life enjoyment*,” these two lines were classified by all of our coders as Income Allocation and Statements.

## Statistical approach

In order to handle the repeated and clustered nature of the experimental design, we employed a mixed fixed and random effects linear model to analyze the data. Each subject lived eight lifetimes, having the opportunity to chat with others in her chat/observation group seven times. During the experiment, each subject chose 72 times (8 lives × 9 periods) how to allocate her income between health and life enjoyment investments. The empirical model takes into account the lack of independence among observations within and among individuals in groups. To do this, the model estimates the fixed effects of lifetime, experimental treatment, and interaction terms, while assessing the random effects for chat group and individual.

## Results

### Descriptive statistics

Descriptive statistics for the main variables to be analyzed are presented in Table 3. For each of the eight “lives” in the experiment, the table shows the means for total enjoyment purchased and the number of strategic queries and advice made per subject during the rest phase following that life during which chat was allowed. *Total* enjoyment purchased is the sum of the amount purchased in each of the nine periods and is proportional to the actual amount the subject is paid. Those data are presented in three columns. The first column shows the means for the treatment group in which each subject's rewards from investments in life enjoyment are *independent* (that is, the rewards are unaffected by the behavior of other subjects in the chat group). There are two columns for the other treatment group in which rewards are *interdependent*. The first of those columns (column 2) presents the counterfactual independent rewards (for comparability purposes) that the subjects in the interdependent chat groups would have received if their rewards were independent. The second of those columns (column 3) presents the rewards they actually received from their investments, after their interdependence is taken into account through the conformity multiplier. It is evident from the table that for both treatment groups, Total Enjoyment Purchased increases with each life, indicating that their performance increasingly approached the optimal investment profile across lives. It is also evident that subjects in the interdependent rewards treatment group achieved increasingly high levels of conformity across lives to maximize the multiplier on their investments. The regression models discussed below will examine these effects in detail.

**Table 3:** **Descriptive statistics**.

**Life**	**Total enjoyment purchased**			**Strategic queries**		**Strategic advice**	
	**Independent**	**Interdependent w/o conformity**	**Interdependent with conformity**	**Independent**	**Interdependent**	**Independent**	**Interdependent**
1	940	1050	1026	0.6	2.7	2.8	8.1
2	1244	1303	1567	0.9	2.5	2.1	11.4
3	1400	1436	1830	0.4	2.3	1.9	8.4
4	1587	1543	2052	0.2	2.8	1.9	7.8
5	1645	1557	2138	0.3	1.7	1.4	6.6
6	1679	1645	2293	0.4	1.3	1.4	5.5
7	1759	1633	2303	0.1	1.0	1.0	5.5
8	1764	1705	2427	n/a			

The last four columns of Table 3 gives the descriptive statistics derived from the coding of the chat that occurred during rest phases between lives. In contrast to earnings which increase over the course of the experiment, chat queries and advice about strategy are more frequent following the first few lives, and then decrease. Interestingly, advice is about four times more common than queries.

Tables 4, 5 present the results of mixed effect regression model, with fixed effects for the experimental variables and random effects for individual and chat/observation group variables as explanatory variables of strategic queries and advice, respectively. In both tables, Model I regresses the dependent variable on the experimental variables life, experimental treatment (Independent vs. Interdependent rewards), and the interaction terms that represent the relationship between the experience and strategic chat. The random effects of this mixed effect model allow us to control for the influence of individual subject and chat/observation group. There are strong effects of life and treatment on both queries and advice, and some of the interaction terms are significant as well. The interdependent groups both made more queries and gave more advice than those with independent rewards, as would be expected by the gains from coordination and conformity. Relative to the last lives, strategic chat of both types was greatest early in the experiment when learning and behavior change was greatest. Rates of decline in chat during the course of the experiment were similar, but there were small significant interactions between life and experimental treatment are weak, but significant; as a result they are included in the model.

**Table 4:** **Predictors of strategic queries**.

	**df**	**Model 1**		**Model 2**	
		**Estimate**	**Sig**.	**Estimate**	**Sig**.
**PARAMETER**					
Intercept	274	0.26	0.000	0.22	0.014
Life 1^†^	924	0.41	0.000	0.41	0.000
Life 2	924	0.36	0.000	0.36	0.000
Life 3	924	0.31	0.001	0.31	0.001
Life 4	924	0.43	0.000	0.43	0.000
Life 5	924	0.16	0.097	0.16	0.097
Life 6	924	0.07	0.477	0.07	0.477
Independent Rewards^‡^	274	−0.25	0.036	−0.25	0.036
Life 1 ^*^ Ind. Rewards^~^	924	−0.26	0.071	−0.26	0.071
Life 2 ^*^ Ind. Rewards	924	−0.16	0.277	−0.16	0.277
Life 3 ^*^ Ind. Rewards	924	−0.23	0.108	−0.23	0.108
Life 4 ^*^ Ind. Rewards	924	−0.4	0.006	−0.4	0.006
Life 5 ^*^ Ind. Rewards	924	−0.1	0.490	−0.1	0.490
Life 6 ^*^ Ind. Rewards	924	0.01	0.971	0.01	0.971
Within group rank				0.02	0.406
**COVARIANCE PARAMETERS/RANDOM EFFECTS**					
Residual		0.4	0.000	0.4	0.000
Chat/obs. Group		0.02	0.101	0.02	0.104
Subject		0.05	0.000	0.05	0.000

† *Life parameters are measure against baseline of Life 7*.

‡ *Interdependent rewards are measured against socially interdependent rewards*.

~ *Interaction terms are measured against baselines of socially interdependent rewards and life 7*.

**Table 5:** **Predictors of strategic statements**.

	**df**	**Model 1**		**Model 2**	
		**Estimate**	**Sig**.	**Estimate**	**Sig**.
**PARAMETER**					
Intercept	76	1.38	0.000	1.98	0.000
Life 1^†^	924	0.65	0.008	0.65	0.008
Life 2	924	1.48	0.000	1.48	0.000
Life 3	924	0.72	0.004	0.72	0.003
Life 4	924	0.58	0.018	0.58	0.018
Life 5	924	0.28	0.247	0.28	0.244
Life 6	924	0.00	1.000	0.00	1.000
Independent Rewards^‡^	274	−1.13	0.013	−1.13	0.013
Life 1 ^*^ Ind. Rewards^~^	924	−0.21	0.578	−0.21	0.576
Life 2 ^*^ Ind. Rewards	924	−1.20	0.001	−1.20	0.001
Life 3 ^*^ Ind. Rewards	924	−0.50	0.182	−0.50	0.180
Life 4 ^*^ Ind. Rewards	924	−0.36	0.334	−0.36	0.331
Life 5 ^*^ Ind. Rewards	924	−0.18	0.626	−0.18	0.624
Life 6 ^*^ Ind. Rewards	924	0.10	0.782	0.10	0.780
Within group rank				−0.24	0.000
**COVARIANCE PARAMETERS/RANDOM EFFECTS**					
Residual		2.64	0.000	2.62	0.000
Chat/obs. Group		1.03	0.001	1.06	0.001
Subject		0.71	0.000	0.60	0.000

† *Life parameters are measure against baseline of Life7*.

‡ *Interdependent rewards are measured against socially interdependent rewards*.

~ *Interaction terms are measured against baselines of socially interdependent rewards and life 7*.

**Figure 6:**
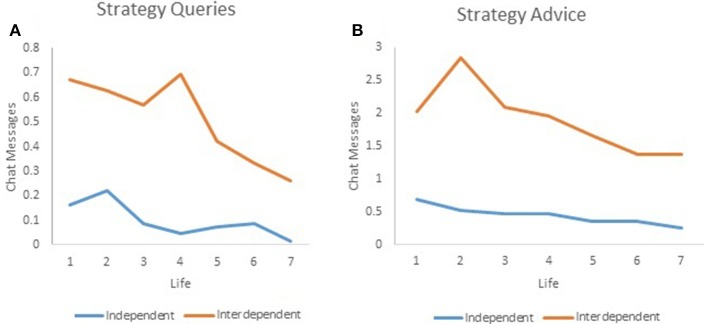
**(A)** Strategy queries. **(B)** Strategy advice.

These effects can be seen clearly in Figures 6A,B, which plot the expected marginal means derived from the Model 1 regressions presented in Tables 4, 5, respectively. On average, queries about investment strategy are about five times more frequent and advice is about 4 times more common in the interdependent than independent rewards treatment. This is to be expected, given that advice and queries that increase conformity of investment in life enjoyment have direct monetary payoffs for those in the interdependent rewards treatment. However, it is also interesting to note that in both treatments, advice is about four times more common than are queries. This is particularly interesting in the case of the independent rewards treatment, because subjects are providing this even though they do not get any direct monetary benefits from giving advice.

If strategy advice is given to help fellow group members, we would expect that higher earning individuals would be more likely to give advice. To test this, Model 2 in Tables 4, 5 adds one additional explanatory variable to the base Model 1, *within-group earnings rank*. This variable, with levels one to four, ranks each of the four members of the chat/observation group in terms of how much they earned in the life previous to that chat session (with one being the highest, and four being the lowest earner). The results show that *high earners are more likely to give advice*, with advice statements decreasing by about 0.24 with each successive rank. However, rank did not have a significant effect on queries, which were less common than advice.

The last set of rows in Tables 4, 5 present the random effects, the residual unaccounted for variance and the effects due to individual subject and chat group. Some of the unobserved characteristics of individual subjects and chat groups may be related to personalities, their willingness to share their knowledge, and the dialog dynamics that are generated within a chat group. In the case of queries, there were significant random effects in subject's play, but the effects of chat group were not significant. In the case of strategic advice, both individual and chat group random effects were significant, and in fact, the random effects estimate for chat group was slightly greater than for subject.

Together, these results support predictions 4–7 above: Subjects engaged in helpful chat in both treatments (P4); there was significant heterogeneity among both subjects and groups in chat frequencies (P5); chat was most common early in the experiment (P6); and chat was more frequent in the interdependent treatment (P7).

### Predictors of enjoyment earnings and assessment of social effects

Due to the dramatic differences in chat by treatment and the expectation that the variance within and among chat groups would differ between the two treatments, we analyze the Enjoyment Earnings for the two treatment groups, separately. Similar to the previous analysis, we use a mixed effects regression models to analyze the determinants of life enjoyment, strategic chat and life enjoyment. Tables 6, 7 present the results for the independent- and interdependent- rewards treatments, respectively. Model 1 presents the baseline model in which Enjoyment Earnings are regressed on life alone. Model 2 adds an additional variable, Total Strategic Chat, to examine whether verbal exchanges over strategy improved earnings in the next life. Total Strategic Chat is the sum of both queries and advice statements over all four members of the group following a given life.

**Table 6:** **Enjoyment earnings without socially dependent rewards**.

	**Model 1**			**Model 2**		
	**df**	**Estimate**	**Sig**.	**df**	**Estimate**	**Sig**.
**PARAMETER**						
Intercept	39	1764	0.000	36	1761	0.000
Life 1	469	−824	0.000		Δ	
Life 2	469	−520	0.000	407	−526	0.000
Life 3	469	−364	0.000	405	−369	0.000
Life 4	469	−176	0.000	403	−179	0.000
Life 5	469	−119	0.004	402	−122	0.003
Life 6	469	−85	0.037	402	−86	0.031
Life 7	469	−5	0.896	402	−7	0.860
Total strategic chat				397	2	0.638
**COVARIANCE PARAMETERS/RANDOM EFFECTS**						
Residual		56,204	0.000		53,691	0.000
Chat/obs. Group		35,053	0.000		41,606	0.000
Subject		10,817	0.167		11,095	0.199

*Δ Because Chat periods only occur after Life 1, Model 2 does not include Enjoyment Earnings for life 1*.

**Table 7:** **Enjoyment earnings with socially dependent rewards**.

	**Model 1**					**Model 2**				
	**df**	**Interdependent w/o conformity**		**Interdependent with conformity**		**df**	**Interdependent w/o conformity**		**Interdependent with conformity**	
		**Estimate**	**Sig**.	**Est**.	**Sig**.		**Est**.	**Sig**.	**Est**.	**Sig**.
**PARAMETER**										
Intercept	32	1705	0.000	2427	0.000	31	1698	0.000	2390	0.000
Life 1	609	−655	0.000	−1401	0.000		Δ			
Life 2	609	−402	0.000	−860	0.000	522	−406	0.000	−882	0.000
Life 3	609	−269	0.000	−597	0.000	523	−276	0.000	−639	0.000
Life 4	609	−162	0.000	−375	0.000	522	−166	0.000	−399	0.000
Life 5	609	−148	0.000	−289	0.000	522	−152	0.000	−310	0.000
Life 6	609	−60	0.080	−134	0.006	522	−62	0.055	−143	0.002
Life 7	609	−72	0.036	−124	0.011	522	−72	0.025	−124	0.007
Total strategic chat						541	1	0.473	5	0.005
**COVARIANCE PARAMETERS/RANDOM EFFECTS**										
Residual		51,678	0.000	102,943	0.000		45,205	0.000	92,560	0.000
Chat/obs. Group		11,533	0.000	10,179	0.013		13,567	0.000	14,248	0.003
Subject		42,449	0.003	133,297	0.002		51,138	0.003	157,171	0.002

*Δ Because Chat periods only occur after Life 1, Model 2 does not include Enjoyment Earnings for life 1*.

For the independent rewards treatment, Table 6 shows that for the base Model 1 earnings increase by almost 90% during the course of the experiment from 940 in life 1 to 1764 in life 8. Model 2 shows that Total Strategic Chat did not have a significant effect on earnings. However, the random effects terms do show appreciable group level random effects, suggesting that observing other group members' play and/or the chat did have effects on behavior. Nevertheless, the estimates for random effects at subject's level were a little more than three times as high as for chat groups (35,053 vs. 10,818).

Table 7 presents the results of the estimation of the regression models for the interdependent treatment: both for the counterfactual earnings without the conformity multiplier and the actual earnings, taken into account the conformity effect. From the Model 1 analysis, we see that earnings also increase from life to life, starting from a mean of 1050 in life 1 and ending with mean of 1704 in life 8 without the conformity multiplier, and from 1026 to 2427 with the multiplier. Adding Total Strategic Chat in Model 2, we see that it has no significant effect on earnings without the multiplier, but a large effect with the multiplier.

Taken together, the results in Tables 6, 7 suggest that we cannot detect an effect of chat on solving the dynamic problem of optimizing investments over the nine-period life course, but we can detect an effect of chat on improving earnings through the conformity multiplier. In other words, subjects were able to coordinate their strategies and make similar investments in each period; the chat appears to have facilitated this coordination.

**Figure 7:**
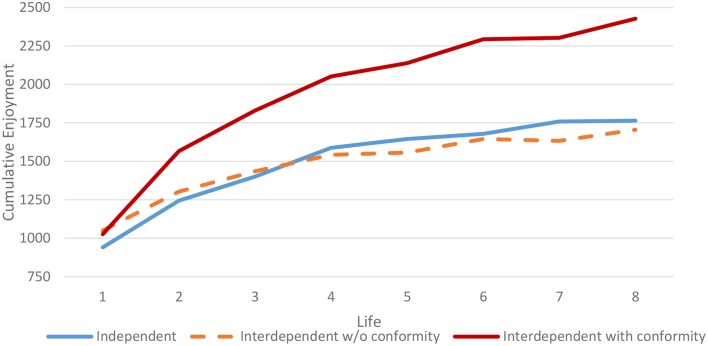
**Cumulative lifetime enjoyment, expected marginal means**.

Figure 7 illustrates these effects by plotting the expected marginal means for enjoyment earnings from the Model 1 regressions in Tables 6, 7. Earnings for both treatments increase with each progressive life, and are very similar on average for the two treatments, when the conformity bias is not taken into account. However, the interdependent chat groups also increasingly took advantage of the conformity multiplier (as can be seen by the increasing distance between the red and orange lines).

The dramatic effects of introducing interdependence in rewards can be seen from the variance decomposition of the random effects. As opposed to the independent rewards case where the chat group random effects were one third as large as the individual subject random effects, they were 13 times larger (133,297 vs. 10,179) in the case of actual enjoyment earnings in the interdependent case. The ratios of the within and between group variances reverse moving from independent to interdependent rewards. The differences between the two treatments were highly significant (*p* < 0.0001), for both within-group variance (higher in the independent rewards treatment) and between-group variance (higher in the interdependent treatment).

**Figure 8:**
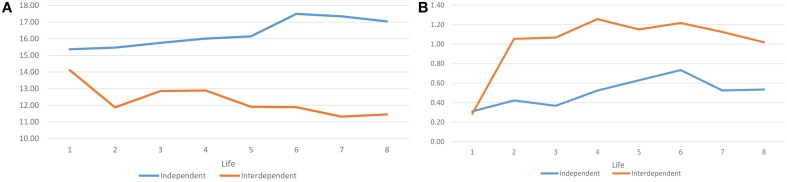
**(A)** Mean standard deviation of enjoyment investment within groups. **(B)** Standard deviation of the mean enjoyment investment among groups.

Those effects are illustrated in Figure 8A shows the mean standard deviation of enjoyment investment within groups for the two treatments. It demonstrates the dramatic reduction in within group variance in investments in the interdependent-rewards treatment. By life 2, subjects have managed to coordinate their investments appreciably, with the lowest variances to be found in lives 7 and 8. Figure 8B shows the standard deviation of mean investments among groups (for each period and each group, the mean of investments is first calculated, and the standard deviations of those means among groups are calculated). Here, the dramatic increase in variance among groups due to the conformity effect in the interdependent treatment is also visible by life 2.

Finally, to ask whether the conformity effect might lead some groups to converge on suboptimal strategies, we examined the likelihood of being in the bottom quartile of earnings at the level of the chat group. To make the data comparable, we used the total enjoyment purchased for the groups in each treatment, ignoring the conformity multiplier. Those results are presented in Table 6, using data from lives 7 and 8 when earnings in both treatments were highest.

Table 8 shows that just over a third of the chat groups in the interdependent rewards treatment were in the lowest quartile of mean earnings (15/44), whereas only 11% were in the lowest quartile in the independent treatment, leading to an odds ratio of about 3. These results suggest that the focus on social conformity in investment in life enjoyment not only increased variance among groups, but also resulted in some groups stabilizing at behavioral strategies quite far from optimal dynamic performance.

**Table 8:** **Cumulative earnings in the lowest quartile**.

**Lowest quartile enjoyment earnings**	**Treatment group**		**Total**
	**Independent**	**Interdependent**	
No	30	29	59
Yes	4	15	19
Total	34	44	78

**Figure 9:**
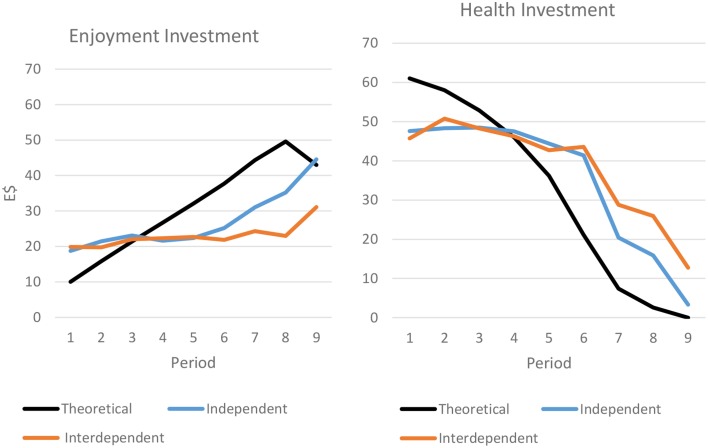
**Observed and theoretically optimal investment strategies**.

Figure 9 examines this possibility by comparing observed investment behavior with optimal investment behavior. A visual inspection of the figure suggests that subjects in the independent rewards treatment converged more on the optimal strategy on average than those in the interdependent treatment. Unlike the theoretical optimum, investments in life enjoyment in the interdependent treatment tend to remain flat rather than increase throughout life, and investments in health decrease much less than is optimal.

Together, these results support predictions 1–3 with the interdependent treatment decreasing within-group variance (P1) but increasing between-group variance (P2), more frequently resulting in behavior far from the optimum with respect to the dynamic problem (P3).

## Discussion and conclusions

These findings support the central hypothesis motivating this paper: incentives for conformity promote prosocial behavior, but also increase variance among groups in equilibrium outcomes, leading to a higher probability of convergence on suboptimal strategies. This tension between gains from conformity and individual optimization may help explain the striking variability in health behavior across regions, ethnic groups and socioeconomic strata. The social costs and benefits of cigarette smoking, alcohol consumption, physical exercise, and eating patterns are likely to vary with respect to their frequency in the networks in which individuals are embedded. From a social perspective, overweight or over-exercise, for that matter, may be relative terms.

These findings are related to the growing literature on social network effects on health and on the growing obesity epidemic. A particularly related paper (Strulik, 2014) concerns how exogenous shifters in the effective costs of food interact with social effects to generate progressive effects on health that are longer term and larger than would be expected from economic considerations alone. Focusing on the fact that increases in obesity postdate by more than a decade the decreases in the costs of food, the paper presents a theoretical model that posits a trade-off between the enjoyment utility of eating and the social disutility of being overweight and being considered less attractive. It shows that an exogenous shifter in the costs of food, which would increase food consumption at the optimum, would at first generate a small increase in overweight that would becoming increasingly larger through time due to social effects. As people gain weight, the social disutility of being fatter than the average shifts with the shifting average. That paper also shows that the exogenous shifters can interact with SES effects on food and non-food consumption to increase SES differences in obesity. Our results suggest that “cultural” differences in norms will also interact with such economic considerations to possibly generate even greater variance among groups and social networks.

These results are also consistent with the growing body of evidence on social network effects on health-related behavior. For example, while there is strong evidence of “hemophilic” self-selection in social network formation and maintenance with like types assorting together (e.g., Christakis and Fowler, 2007; Hutchinson and Rapee, 2007), there is also evidence that experimentally-induced random assignment to networks can influence behavior as well. For example, Girard et al. (2014) observed naturalistic network formation among college students, but also experimentally induced network ties among students. They found that students' formation of social networks was influenced by both homophilic considerations and the characteristics of the partners with whom they were randomly assigned. Regional and ethnic variation in health behavior probably reflects both the random effects of the social network one is born into and subsequent self-selection processes. Future experiments will allow us to combine random and endogenous partner formation in controlled ways to determine their relative effects. Another related paper (Ajilore et al., 2014) utilizes spatial econometrics to decompose social effects on weight outcomes into social “multiplier” (enhancement) and social norm effects, respectively; they define the social multiplier effect as “change in behavior of any one individual in the peer group spreads to the other members of the group, (whereas) with the social norm effect, individuals conform to the average behavior of the entire peer group.” They found stronger effects of norms on BMI and of social multiplier on overweight status. Our experiment combines those two effects in the conformity multiplier. However, in our experimental context, future work can analyze multiplier and normative effects separately through different rewards functions.

A recent study (Daw et al., 2013) found that the serotonin transporter–linked polymorphic region (5HTTLPR), a gene associated with environmental sensitivity, moderates the association between smoking and drinking patterns at adolescents' schools and their corresponding risk for smoking and drinking themselves. They concluded that an individual's susceptibility to school-level patterns of smoking or drinking is conditional on the number of short alleles he or she has in 5HTTLPR. This result suggests that one fruitful future direction for experimental laboratory-based research might be to combine genetic analysis of allele frequencies of subjects with both changing dynamic optimization problems and social manipulations.

All of our subsidiary predictions (4–7) regarding chat, were supported. Helpful chat was common in both treatments, with significant heterogeneity among groups and most chat being concentrated early in the experiment. Strategic chat was much more frequent in the interdependent rewards (conformity) treatment.

In the independent-rewards treatment, there were no monetary incentives for subjects to help (or mislead) others in finding the optimal strategy of investments, nor were subjects given instructions about what they could discuss between lives. About one third of chat messages communicated concerned harvesting strategies, while two thirds were directed toward optimal investment (about 2 messages per life). Most messages delivered advice rather than a query concerning strategy. Individual monetary incentives might explain queries, but are less likely to explain advice. A subject who earned more than others in her chat group was more likely to provide strategic advice, but was no more likely to make a query. This suggests that better earners were intrinsically motivated to help those who did worse.

The interdependent rewards treatment provided a strong extrinsic monetary incentive for subjects to coordinate on behavior. This motivation resulted in both absolutely more strategy chat and a greater relative emphasis on strategy than on other topics. People in the interdependent treatment sent about four times as many strategy messages as the independent rewards treatment, but they sent fewer messages about topics outside the experiment (1.2 vs. 3 messages on average per life per chat group). Just as in the independent treatment, higher earners in the interdependent dependent treatment offered more strategic advice than lower earners, and advice was much more common than queries.

With respect to the impacts of chat on dynamic optimization, results were largely negative. There were no significant effects of chat quantity on earnings in the independent-rewards treatment. In the case of the interdependent rewards, we also found no significant effects of chat on earnings without taking into account the conformity multiplier. However, actual earnings, taking into account the multiplier, were positively associated with the number of strategy chat messages sent in a life. Chat appears to have facilitated conformity to one strategy, rather than investment optimization over the life course.

The dynamic optimization problem subjects faced was particularly complex and non-linear. They showed through their behavior that they were able to improve their performance over time, but it may have been difficult to put those improvements into words in simple chat messages, so quantity of messages without reference to quality is a poor measure of information flow. Nevertheless, significant social learning still appears to have occurred, given the significant random effects of chat groups on earnings and investments in both health and life enjoyment.

Perhaps, the gains from conformity can compete with other strategic problems individuals face, more generally. In the face of uncertainty, doing what most others do (positive frequency dependent modeling) can often be the best strategy, since it integrates information across individuals and over time (Boyd and Richerson, 1988; Henrich and McElreath, 2003). Moreover, activities that provide enjoyment utility at a potential cost to health, such as smoking, alcohol consumption, and excessive eating, are often done in social contexts. Thus, the variant individual who chooses to avoid those activities and invest in health and a substitute activity will forego opportunities for social exchange at the same time. It is likely that the extrinsic monetary payoff for social conformity imposed by our experiment does not adequately model the intrinsic emotional, moral and status-related dimensions of social conformity motivating individual behavior. It may not be surprising that this experimental intervention decreased within-group variance. However, it is striking that the intervention dramatically increased among group variance, as predicted by our hypothesis. There was no extrinsic monetary payoff for among group variance; in fact, variance away from the optimal strategy was economically costly.

The processes generating varying social equilibria in health behavior and status merit further investigation. Behavioral economic experiments that focus on the interplay of dynamic optimization problems and social forces are likely to provide new insights into why so many different equilibria are observed, and may be particularly productive in explaining changing patterns of health. For example, the question of heterogeneous skill/income levels would pose a serious problem when strong social norms are in place. In the experiments reported, after a few periods of harvesting experience, subjects became very good at harvesting near optimally. Furthermore, in our experiments, the naive harvesting policy (take the next target whatever it is) performed 97% efficiently if implemented with perfect vigilance. Therefore, differences in income due to heterogeneity in skill levels were minimized. This was exactly as we intended; the task required vigilance and real effort but resulted in low variance in subject disposable income. This made joint decision-making in the social conformity treatment less subject to the complexity that results with group member wealth disparities. Most inefficiency, and there was not a lot, was simply due to lack of vigilance. Experimental environments can easily be created to allow for a controlled experimental environment in which subjects' inherent skill sets (e.g., hand/eye coordination) can produce heterogeneous income profiles. We will run that experiment after constructing an environment that also provides a mechanism for conformity groups to form and reform endogenously. That more complicated environment will be implemented in the next generation of experimentation.

Many other validity issues can be addressed under controlled laboratory conditions. For example, lifetime length duration and individual discounting issues will be considered by employing a much extended time horizon (web-based experiments that run months rather than hours), a much higher number of decision points during any lifetime, and the real temptation to abscond with continuously available electronic payoffs for the purpose of real life enjoyment during the course of the experimental lifetime. Individual risk attitudes toward health preservation will become influential when we introduce stochastic shocks to health and the possibility of purchasing reparative insurance. Social impingement on health-creating activities will be implemented through tedious real labor tasks that can be abbreviated through subject coordinated on-line co-participation. Similarly, subject arranged simultaneity of on-line activity will be used to amplify enjoyment derived from leisure tasks (browsing). Understanding what we have observed and discovered in the experiments reported in this article will allow us to directly confront many validity questions in the next generation of laboratory controlled health decision experiments.

### Conflict of interest statement

The authors declare that the research was conducted in the absence of any commercial or financial relationships that could be construed as a potential conflict of interest.

## Footnotes

^1^For a complete description of the revenue possible and the optimal harvesting strategy see Supplementary Material.

^2^The second derivatives are calculated in the Supplementary Material.

^3^Health degeneration d_t_ = {−16, −17, … −23, −24}.

^4^Rev is a fixed parameter that indicates participant harvesting proficiency. See Supplementary Material for further discussion.

^5^The parameters in the environments we designed were such that the optimal B^*^_t_ was rarely anything other than 0. This considerably reduced the dimension of the decision making problem faced by participants. On rare occasions, during the move from harvesting to retirement (periods 6–7), there was a minor improvement in overall life enjoyment by banking some harvest income to smooth investment in life enjoyment.

^6^Supplementary Material provides the example of period 9.

^7^This is true for all periods except where a boundary condition is met (only in periods 8 and/or 9) and the marginal return on any investment in health is dominated by investment in life enjoyment (L_t_) so the optimal investment in health is zero (I_t_ = 0).

^8^There are only 2 periods in the 36 displayed where it behooves subjects to bank some earned revenue for the purpose income smoothing: in period 8 of the Flat No Retirement regime where health will fall precipitously in period 9, and in period 6 of the Tiered Retirement regime where income falls precipitously in period 7, the first period of retirement.

^9^Four coders had classified the chat lines of the Independent treatment. To be consistent with the classification of chat in the Interdependent treatment, we used a classification based on those coders with inter-reliability rates similar to the coders of the *Interdependent* treatment. The classification manual provided to coders is presented with the additional materials.
