# Supplementary material for: Dynamic optimization and conformity in health behavior and life enjoyment over the life cycle
Source: Front Behav Neurosci. 2015 Jun 16;9:137. doi: 10.3389/fnbeh.2015.00137 (PMC4468389; doi:10.3389/fnbeh.2015.00137)
Supplement: Supplementary file 1 [file DataSheet1.DOCX]

**Appendix 1: Real Effort Harvesting**

During the first part of each period in each life, participants were required to undertake a real-effort harvesting task in which the participant could earn revenue, R_t_, that she could subsequently invest in either life enjoyment (a cash reward) or health preservation for subsequent periods of the current lifetime. The amount of time allowed for the harvesting task during each period (maximum 30 seconds) was directly proportional to the participant’s current level of health, H_t_, (between 0 and 100), so investment in upgrading health enabled a higher levels of harvesting in future periods. The initial health condition, 85, and the natural degeneration of health across all periods of life {-16, -17, … -23, -24} were preprogrammed and identical for all participants in all lifetimes in all experimental treatments. The health degeneration occurred after harvesting just before investment for the current period began. Never investing in health would result in the participant dying (not being able to continue to harvesting and investing) in period 5.

The harvesting task assigned to participants required vigilance and some manual dexterity but was designed so that most participants could perform at a high enough level that their harvest earnings and optimal investment strategies would be quite comparable. The task involved a sequence of 30 targets that would skirt across a circular harvesting field. Each target had a one of four different harvest values, and each target took two seconds to skirt across the field, after which it disappeared. To harvest the target the participant simply needed to click on the harvesting field while that target was viable. Once a click was made it would take 2 seconds to process the harvested target during which time the participant could harvest no other targets although she could see the unavailable targets as they skirted by. If the participant’s current health were at level H_t_ ∈[0,100], then during the first 30x(100- H_t_)/100 seconds of the harvest period she would see targets go by that she was unable to harvest due to her deteriorated health. Similarly, if a target were only partially processed by the end of the previous period, processing would complete at the beginning of the next period adding a small increment to any downtime due to deteriorated health.

The following table shows the target values available for harvest during each period of each life, and the probabilities that each target would be the next to arrive:

| **Period Target Vector** | **Target Probabilities** | **Expected Income** |
| --- | --- | --- |
| {13*, 10*, 8*, 6} | {.22, .29, .31, .18} | R_t_* = 94 (H_t_/100) |

The optimal harvesting strategy was simply to harvest either the three most valuable targets whenever one became available and always ignore lower valued targets. If the participant implemented the optimal harvesting policy during any particular harvesting period t, and had a current health level of H_t_, then R_t_* was her expected optimal harvesting revenue.

For any given 30 second period the actual harvest revenue can vary slightly about R_t_* even if the optimal harvesting policy is applied, depending upon the random arrival sequence of the various targets. Furthermore, the skill level (hand eye coordination and required vigilance) of any participant in applying the optimal harvesting policy can reduce the expectation of revenue from harvesting. A perfectly skilled harvester who has a particular proportion, H_t_/100, of harvesting time available in a given harvesting period because of his current health, collects expected revenues rev (H_t_/100) where rev = 94. Because the revenues collected from harvesting become income available to invest in health and life enjoyment, lesser harvesting skill, rev < 94, can have a significant effect on the optimal investment plans for participants of varying skill.

To compute the optimal harvesting strategy, consider, for example, the target set V= {13, 10, 8, 6} where the probability of encountering each target type during the next second is given by p={.22, .29, .31, .18}. Given it takes 2 seconds of handling time to process any target, the harvest value per second for each type of target is given by V/2 = {6.5, 5, 4, 3}. The total value per second derived by harvesting only the n most valuable targets is given by ∑v_i_p_i_ = {2.86, 5.76, 8.24, 9.32}. The total handling time for the n most valuable targets is given by 1+ ∑2p_i_ = {1.44, 2.02, 2.64, 3}. And finally, the total value per second of total handling time is given by ∑v_i_p_i_ / (1+ ∑2p_i_) = {1.99, 2.85, 3.12, 3.1}. The optimal harvesting policy is to always take whichever of the first three targets shows up next. In a 30 second harvesting period this policy would generate a total harvest value of 30 x 3.12 = 94 units of value.

The parameters in the experiment are set such that rev= 94 is the expected revenue per period for a perfectly skilled harvester who is 100% healthy. In the experiments reported, the participants displayed mean harvesting skills that were less than perfect (rev= 87), but with low variance. Because the revenues R_t_ collected from harvesting become the income available to invest in health and life enjoyment, lesser harvesting skill can have a significant effect on the optimal investment schedule. We use rev= 87, the mean harvesting skill of all subjects, as the baseline parameter for computing optimal investment planning throughout this paper.

**Appendix 2: Computation**

The transition equation relating health at the end of period t to health at the end of period t-1 is given by:

$${H_{t}=Min [100, H}_{t-1}-d_{t}+30\frac{1-ⅇ^{-.025I_{t}}}{1+ⅇ^{-.025I_{t}}} ]$$

The first derivative of health w.r.t. investment in health is given by:

$$\frac{dH_{t}}{{dI}_{t}}= 30\cdot.025ⅇ^{-.025I_{t}} \left[ \frac{1}{1+ⅇ^{-.025I_{t}}}+\frac{(1-ⅇ^{-.025I_{t}})}{\left( 1+ⅇ^{-.025I_{t}} \right)^{2}} \right]$$

or,

$$\frac{dH_{t}}{{dI}_{t}}= \frac{1.5ⅇ^{-.025I_{t}}}{1+{2ⅇ}^{-.025I_{t}}+ⅇ^{-.05I_{t}}}$$

The second derivative of health w.r.t. investment in health is given by:

$$\frac{d^{2}H_{t}}{{{dI}_{t}}^{2}}= 1.5 \left[ \frac{-.025ⅇ^{-.025I_{t}}}{1+{2ⅇ}^{-.025I_{t}}+ⅇ^{-.05I_{t}}}+ \frac{.05(ⅇ^{-.025I_{t}})(ⅇ^{-.025I_{t}}+ⅇ^{-.05I_{t}})}{{(1+{2ⅇ}^{-.025I_{t}}+ⅇ^{-.05I_{t}})}^{2}} \right]$$

or,

$$\frac{d^{2}H_{t}}{{{dI}_{t}}^{2}}= 1.5 \left[ \frac{-.025ⅇ^{-.025I_{t}}-.05ⅇ^{-.05I_{t}}-.025ⅇ^{-.075I_{t}}}{({1+{2ⅇ}^{-.025I_{t}}+ⅇ^{-.05I_{t}})}^{2}}+ \frac{.05(ⅇ^{-.05I_{t}}+ⅇ^{-.075I_{t}})}{{(1+{2ⅇ}^{-.025I_{t}}+ⅇ^{-.05I_{t}})}^{2}} \right]$$

$$\frac{d^{2}H_{t}}{{{dI}_{t}}^{2}}= -.025 \left[ \frac{ⅇ^{-.025I_{t}}-ⅇ^{-.075I_{t}}}{{(1+{2ⅇ}^{-.025I_{t}}+ⅇ^{-.05I_{t}})}^{2}} \right]$$

The consumption function which gives subject earnings, *E_t,_* from life enjoyment in period t as a function of the portion of harvest and retirement returns that are invested in life enjoyment, L_t_, is given by:

$$E_{t}= 250(1+H_{t}/100)(1-ⅇ^{-.028L_{t}})$$

The first derivative of life enjoyment w.r.t. investment in life enjoyment is given by:

$$\frac{dE_{t}}{{dL}_{t}}= 7(1+H_{t}/100)(ⅇ^{-.028L_{t}})$$

The second derivative of life enjoyment w.r.t. investment in life enjoyment is given by:

$$\frac{d^{2}E_{t}}{{{dL}_{t}}^{2}}= -.196(1+H_{t}/100)(ⅇ^{-.028L_{t}})$$

The final period (9) life enjoyment optimization problem is given by: (note that E_9_ is a function of the final period investment decisions, I_9_ and L_9_, and several pre-determined parameters, B_8_ (what’s remaining in the bank after the previous period 8) and R_9_ (returns from harvesting or retirement in current period 9) and d_9_ (degeneration of health (=24) that occurs in current period 9 before investing)

Maximize: $E_{9}= 250(1+H_{9}/100)(1-ⅇ^{-.028L_{9}})$

Subject to: $B_{8}+R_{9}= I_{9}+ L_{9}$

${H_{9}= H}_{8}-d_{9}+30\frac{1-ⅇ^{-.025I_{9}}}{1+ⅇ^{-.025I_{9}}}$

Which is equivalent to maximizing the unconstrained function of I_9_:

$$E_{9}=250(1+{(H}_{8}-d_{9}+30\frac{1-ⅇ^{-.025I_{9}}}{1+ⅇ^{-.025I_{9}}}) /100)(1-ⅇ^{-.028{(B_{8}+R_{9}-I}_{9})})$$

Taking the first order condition with respect to final health investment, I_9_, and setting equal to 0 we get:

$$\frac{dE_{9}}{dI_{9}}=0=250(100+H_{8}-d_{9}+30\frac{1-ⅇ^{-.025I_{9}}}{1+ⅇ^{-.025I_{9}}}) /100)(-.028ⅇ^{-.028{(B_{8}+R_{9}-I}_{9})}+ 75(\frac{.025ⅇ^{-.025I_{9}}}{1+ⅇ^{-.025I_{9}}}+ \frac{.025ⅇ^{-.025I_{9}}(1-ⅇ^{-.025I_{9}})}{{(1+ⅇ^{-.025I_{9}})}^{2}}) (1-ⅇ^{-.028{(B_{8}+R_{9}-I}_{9})})$$

$$\frac{dE_{9}}{dI_{9}}=0=2.5(100+H_{8}-d_{9}+30\frac{1-ⅇ^{-.025I_{9}}}{1+ⅇ^{-.025I_{9}}})(-.028ⅇ^{-.028{(B_{8}+R_{9}-I}_{9})})+ 75(\frac{.025ⅇ^{-.025I_{9}}+.025ⅇ^{-.05I_{9}}}{{(1+ⅇ^{-.025I_{9}})}^{2}}+ \frac{.025ⅇ^{-.025I_{9}}-{.025ⅇ}^{-.05I_{9}})}{{(1+ⅇ^{-.025I_{9}})}^{2}}) (1-ⅇ^{-.028{(B_{8}+R_{9}-I}_{9})})$$

$$\frac{dE_{9}}{dI_{9}}=0=2.5(100+H_{8}-d_{9}+30\frac{1-ⅇ^{-.025I_{9}}}{1+ⅇ^{-.025I_{9}}})(-.028ⅇ^{-.028{(B_{8}+R_{9}-I}_{9})})+ 150\frac{.025ⅇ^{-.025I_{9}}}{{(1+ⅇ^{-.025I_{9}})}^{2}} (1-ⅇ^{-.028{(B_{8}+R_{9}-I}_{9})})$$

Letting d_9_ = 24, we get:

$$0=(190+{2.5H}_{8}+75\frac{1-ⅇ^{-.025I_{9}}}{1+ⅇ^{-.025I_{9}}})(-.028ⅇ^{-.028{(B_{8}+R_{9}-I}_{9})})+ \frac{3.75ⅇ^{-.025I_{9}}}{{(1+ⅇ^{-.025I_{9}})}^{2}} (1-ⅇ^{-.028{(B_{8}+R_{9}-I}_{9})})$$

$$0={\left( 190+{2.5H}_{8} \right)\left( 1+ⅇ^{-.025I_{9}} \right)}^{2}-2.1\left( ⅇ^{-.028{(B_{8}+R_{9}-I}_{9})} \right)\left( 1-ⅇ^{-.05I_{9}} \right)+ 3.75ⅇ^{-.025I_{9}}(1-ⅇ^{-.028{(B_{8}+R_{9}-I}_{9})})$$

The result is a single variable equation in I_9_ that is soluble by a simple univariate search procedure given the state of the participant (B_8,_ H_8_, R_9_) before her optimal investment decision I_9_^*^ is made. R_9_, harvest revenue is simply a linear function of health in previous periods, either rev_c_H_8_ or rev_c_(H_1_+H_2_+H_3_+H_4_+H_5_+H_6_)/6 where rev_c_ is a constant dependent on the target set available. Clearly, L_9_^*^ = B_8_ + R_9_ - I_9_^*^

The following tables provide the complete optimal decision trajectory for decision makers who are perfect harvesters (rev= 94, given the experiment parameters), and the optimal decision trajectory for decision makers who possess the average harvesting skill (rev= 87) that was demonstrated by our experimental subjects.

|  |  |  |  |  |  |  |  |  |  |  |
| --- | --- | --- | --- | --- | --- | --- | --- | --- | --- | --- |
| Independent |  |  |  |  |  |  |  | Harvest Rate Per Health = 0.94 | |  |
| Period | 0 | 1 | 2 | 3 | 4 | 5 | 6 | 7 | 8 | 9 |
| Investment Health | 85 | 69 | 72.7 | 75.1 | 75.9 | 74.7 | 71.1 | 64.3 | 52.8 | 33.6 |
| End Health | 85 | 89.7 | 93.1 | 94.9 | 94.7 | 92.1 | 86.3 | 75.8 | 57.6 | 33.6 |
| Harvest | 0 | 79.9 | 84.3 | 87.5 | 89.2 | 89 | 86.6 | 81.2 | 71.3 | 54.2 |
| Health Investment | 0 | 68 | 66.1 | 63.3 | 59.1 | 53.2 | 44.6 | 32.3 | 12.9 | 0 |
| Life Investment | 0 | 11.9 | 18.2 | 24.2 | 30.1 | 35.9 | 42 | 48.9 | 58.4 | 54.2 |
| Cash On Hand | 0 | 0 | 0 | 0 | 0 | 0 | 0 | 0 | 0 | 0 |
| Life Enjoyment | 0 | 202.3 | 289.3 | 359.7 | 415.5 | 456.5 | 483.1 | 491.5 | 475.8 | 391.1 |
| Marginal ROR |  | 9.51 | 8.12 | 6.93 | 5.86 | 4.92 | 4.02 | 3.13 | 2.15 | 2.05 |
| % Invest in Health |  | 85 | 78 | 72 | 66 | 60 | 52 | 40 | 18 | 0 |

| Interdependent |  |  |  |  |  |  |  | Harvest Rate Per Health = 0.87 | |  |
| --- | --- | --- | --- | --- | --- | --- | --- | --- | --- | --- |
| Period | 0 | 1 | 2 | 3 | 4 | 5 | 6 | 7 | 8 | 9 |
| Investment Health | 85 | 69 | 71.9 | 73.3 | 72.9 | 70.4 | 65.1 | 56.1 | 41.7 | 17.7 |
| End Health | 85 | 88.9 | 91.3 | 91.9 | 90.4 | 86.1 | 78.1 | 64.7 | 41.7 | 17.7 |
| Harvest | 0 | 74 | 77.3 | 79.4 | 80 | 78.6 | 74.9 | 67.9 | 56.3 | 36.3 |
| Health Investment | 0 | 63.9 | 61.5 | 58.1 | 53.2 | 46.5 | 37.2 | 23.6 | 0 | 0 |
| Life Investment | 0 | 10 | 15.8 | 21.4 | 26.7 | 32.1 | 37.7 | 44.3 | 49.6 | 43 |
| Cash On Hand | 0 | 0 | 0 | 0 | 0 | 0 | 0 | 0 | 6.7 | 0 |
| Life Enjoyment | 0 | 173.7 | 256.5 | 323.9 | 376.2 | 414 | 435.5 | 439 | 398.9 | 309 |
| Marginal ROR |  | 9.99 | 8.60 | 7.38 | 6.31 | 5.30 | 4.34 | 3.33 | 2.47 | 2.47 |
| % Invest in Health |  | 86 | 80 | 73 | 67 | 59 | 50 | 35 | 0 | 0 |
